# Supplementary material for: INO80 exchanges H2A.Z for H2A by translocating on DNA proximal to histone dimers
Source: Nat Commun. 2017 Jun 12;8:15616. doi: 10.1038/ncomms15616 (PMC5472786; doi:10.1038/ncomms15616)
Supplement: Supplementary Information — Supplementary Figures, Supplementary Table and Supplementary References [file ncomms15616-s1.pdf]

Supplementary Table 1: Primer sequences

Purpose	Primer	Oligonucleotide sequence (5'-3')
PCR synthesis of DNA with 601-nucleosome positioning sequence for 70N0, 0N70, and 70N20 nucleosomes using p199-1 or p159-1 plasmid as template.	p199-1 70 FW	CGAGCTCGGTACTCGGGAGCTCGGA
	p199-1 0 RV	CCGAGAGAATCCCGGTGCCG
	p199-1 0 FW	CAGGATGTATATATCTGAC
	p199-1 70 RV	TCACACAGGAAACAGCTATG
	p159-1 70 FW	TTT TCC CAG TCT AGA CGT TG
	p159-1 20 RV	TAC TCG GGT TCA ATA CAT GC
Primers for site specific incorporation of photoreactive dUMP analogs at the specified positions, using nucleosome positioning sequence from p199-1 plasmid as template DNA.	-77	CAAGGTCGCTGTTCAATAC
	-68	GTTCAATACATGCACAGG
	-62/-60	AATACATGCACAGGATGTAT
	-58	GTCGCTGTTCAATACATGCACAGGATG TATATATC
	-33/-30	GCCTGGAGACTAGGGAG
	Upstream primer	CACGACGTTGTAAAACGACGGCCAG
Primers for amplification of *INO80-FLAG* with 100bp flanking sequence from yeast genome.	100bp UP FW	ATG TCA CTG GCA GTT CTA CTC AAT AAG G
	100bp DN RV	TTA ATG TAA ATA ACA CAA TAT G

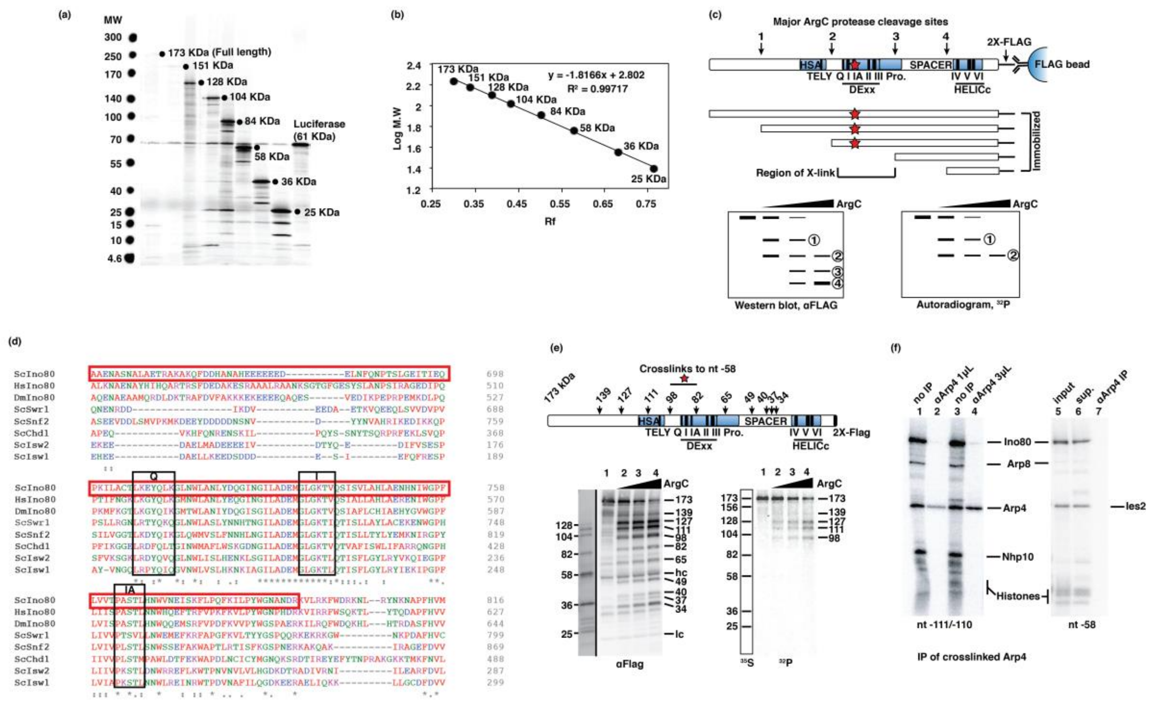

Supplementary Figure 1. Peptide mapping of Ino80 crosslinked to nucleosomal DNA at nt -58 (a) In vitro synthesized [^{35}S]-Methionine labeled peptide markers of different sizes for Ino80-FLAG from the C-terminus, showing their relative migration in 4-20% SDS-polyacrylamide gel against commercial markers of known molecular weights. **(b)** Relative electrophoretic mobility (R_f) of each peptide marker from three technical replicates were calculated and plotted against $\log_{10}$ of their estimated molecular weights to generate a standard curve. Ino80-FLAG fragments resulting from ArgC cleavage were determined from the standard curve. **(c)** Schematic representation of the peptide mapping strategy. Limited ArgC digestion of immobilized Ino80-FLAG generated fragments of different sizes. C-terminal fragments which were retained on the beads after washes were resolved by SDS-polyacrylamide gel electrophoresis. All FLAG-tagged fragments versus those that contained the radiolabel (^{32}P) transferred from DNA were identified by immunoblotting and phosphorimaging respectively. **(d)** Sequence

homology of the region of *Saccharomyces cerevisiae* Ino80 ATPase crosslinked to DNA at nt -58, with Ino80 from other species and ATPase domains of other ATP-dependent chromatin remodelers from yeast. The crosslinked region is highlighted with a red box and conserved helicase motifs within the region are highlighted with black boxes. **(e)** Peptide mapping of the Ino80 catalytic subunit crosslinked to DNA at nt -58, without the addition of ADP in the crosslinking reaction. Numbers in the diagram indicate molecular weights of the C-terminal fragments identified by anti-FLAG immunoblotting (αFLAG image). Sites where proteolysis generates these fragments are indicated along with the locations of conserved domains and motifs in Ino80, and the region crosslinked to DNA. In the immunoblot 'hc' and 'lc' indicate respectively, the antibody heavy and light chains. **(f)** To distinguish between Arp4 and Ies2, which migrate very close to each other on 4-20% SDS-PAGE, Arp4 was immunoprecipitated (IP) from the crosslinked samples (antibody kindly gifted by Dr. Bruce Stillman). Arp4 could be immunoprecipitated from INO80 crosslinked to DNA at nt -111/-110 (lanes 2, 4 versus lanes 1, 3), but not from INO80 crosslinked at nt -58 (compare lanes 5 and 7).

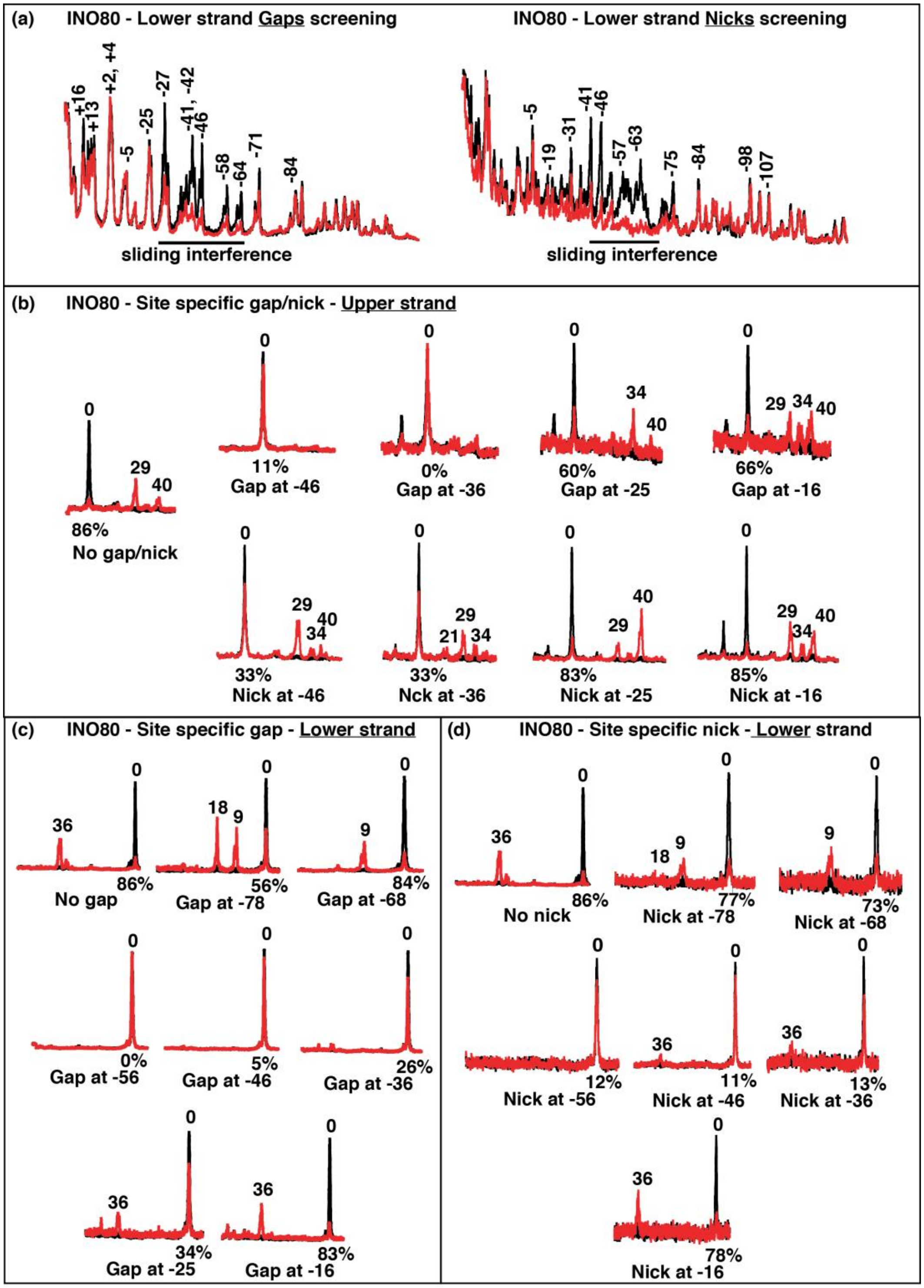

Supplementary Figure 2. Gaps and nicks in DNA block nucleosome mobilization by INO80 (a) The position(s) where gaps or nicks in the lower DNA strand interfere with INO80 remodeling were screened with 0N70 nucleosomes containing random gaps/nicks

as in Figure 3a. **(b)** Nucleosomes with a gap or nick at specified positions placed in the DNA upper strand were remodeled with INO80. Changes in nucleosome position were mapped by site-directed cleavage[59] as in Figure 3b. Nucleosomes with a single gap (c) or nick (d) in DNA at specified positions in the lower strand were remodeled with INO80, and mapped the same as for gaps/nicks in the upper strand. Mobilization of nucleosomes containing site-specific single nucleotide gaps or nicks were performed by adding 800 μM ATP for INO80 or 40 μM ATP for ISW2, and incubating at 30°C for 10 min as in Figure 3. Data are representative of three technical replicates.

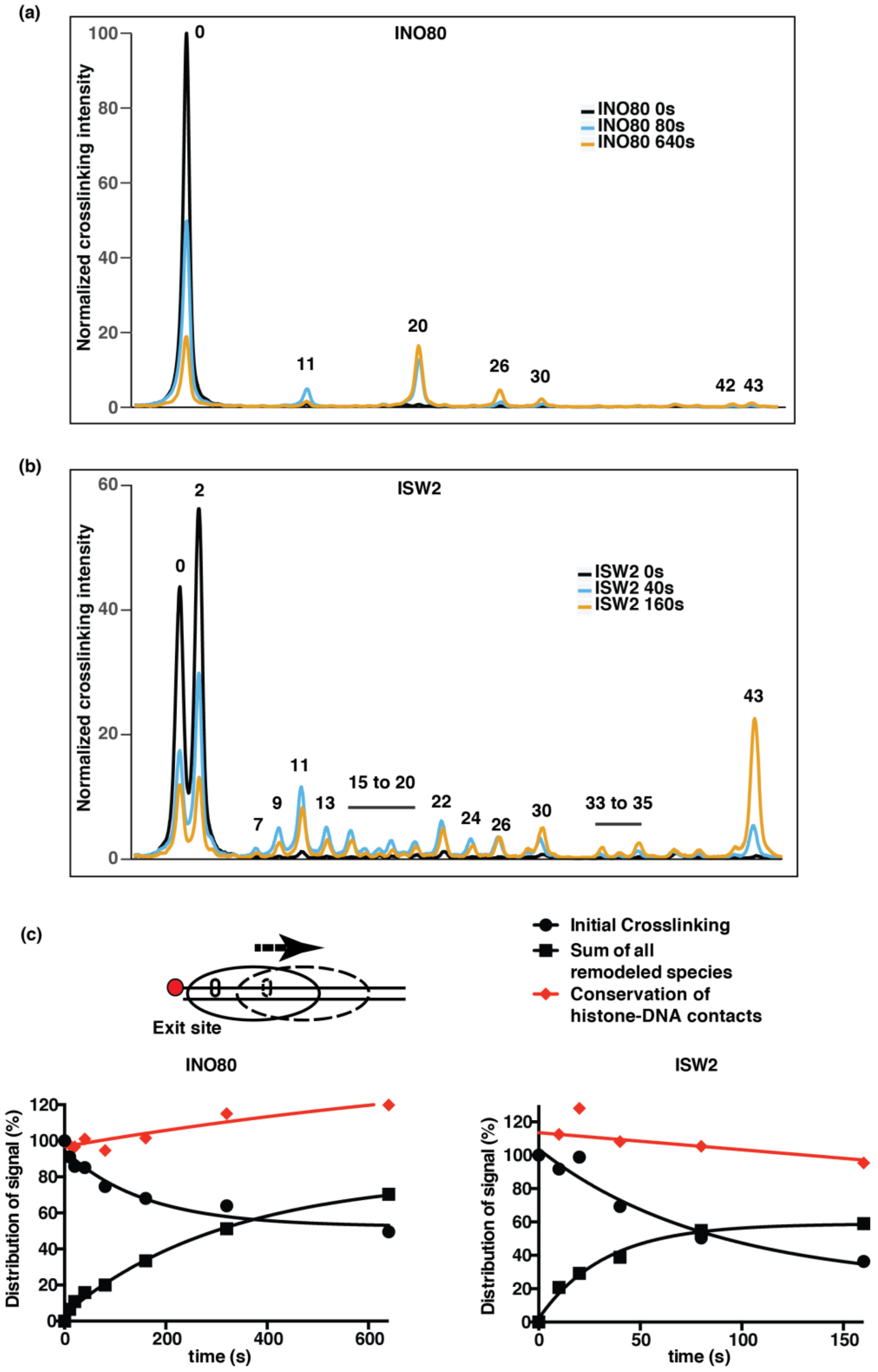

Supplementary Figure 3. INO80 disrupts histone-DNA contacts only at the longer extranucleosomal DNA proximal edge (entry site) of nucleosomes (a-b) Band

intensities of DNA fragments resulting from site-directed mapping of DNA movements across H2B53 at the longer extranucleosomal DNA-proximal edge (entry site) of nucleosomes were overlaid from Figure 4a. Band intensities were normalized to total DNA signal in each lane in Figure 4a. Numbers above the peaks correspond to movements of DNA in nucleotides from the starting position (0). **(c)** DNA movements at the edge of the nucleosome away from the longer extranucleosomal DNA (exit site) were analyzed the same way as for the entry site (as in Fig. 4). The amount of DNA cleaved at the starting position (initial crosslinking), total amount of those bands that accumulate during remodeling (sum of all remodeled species) and conservation of histone-DNA contacts were plotted versus time for INO80 and ISW2 in the same way as for Figures 4b and c. ISW2 and INO80 mediated nucleosome mobilization were initiated with 16 μM ATP and 80 μM ATP, respectively, and stopped with excess EDTA (15 mM); the same as in Figure 4.

(a)

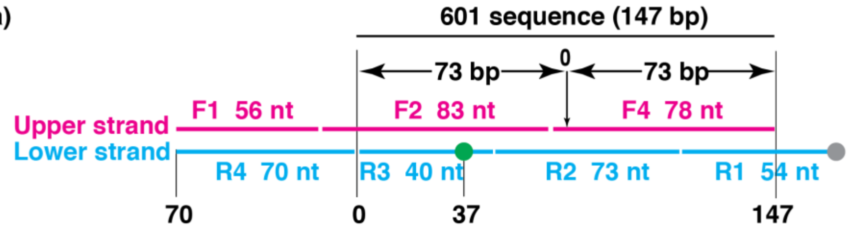

(b)

Upper strand:

5′-AATGACCAAG GAAAGCATGA TTCTTCACACCGAGTTCATC CCTTATGTGA TGGACCCTAT
ACGCGGCCGCATCGGATGTA TATATCTGAC ACGTGCCTGG AGACTTGGGA GTAATCCCCT
TGGCGGTTAA AACGCGGGGG ACAGCGCGTA CGTGCGTTTA AGCGGTGCTA GAGCTGTCTA
CGACCAATTG AGCGGCCTCG GCACCGGGAT TCTCGAT

Lower strand:

5′-/Biotin/GCATGTAAGT GCATGTAAGT ATCGAGAATC CCGGTGCCGA GGCCGCTCAA
TTGGTCGTAG ACAGCTCTAG CACCGCTTAA ACGCACGTAC GCGCTGTCCC CCGCGTTTTA
ACCGCCAAGG GGATTACTCC C/Cy3/AGTCTCCA GGCACGTGTC AGATATATAC
ATCCGAT GCG GCCGCGTATA GGGTCCATCA CATAAGGGAT GAACTCGGTG TGAAGAATCA
TGCTTTCCTT GGTCATT

(c)

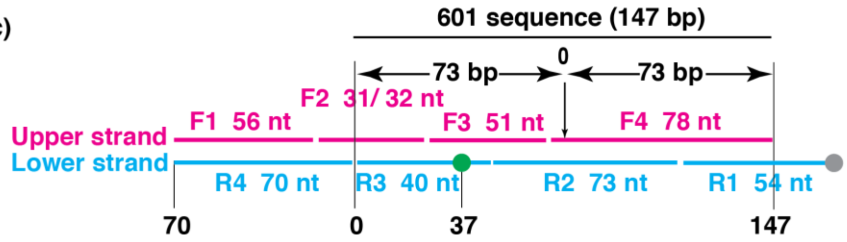

Supplementary Figure 4. Nucleosomal DNA constructs for single-molecule FRET experiments (a) Seven oligonucleotides with overlapping regions were annealed and ligated to generate the template for nucleosome (147 bp) with 70 bp of extranucleosomal DNA (70N0) on one side and 20 nt of single stranded overhang on the other side with a 5´-biotin. A Cy3 molecule (FRET donor) replaced nt -37 in the lower strand. **(b)** Sequence of the upper and lower strands of the DNA construct. **(c)** To introduce a single nucleotide gap or nick at nt -56 from the dyad axis in the upper strand, four oligonucleotides was used instead of three. F2 was 31 nt for gap and 32 nt for nick. F3 did not contain a 5´-phosphate in case of the nick so that F2 and F3 cannot be ligated.

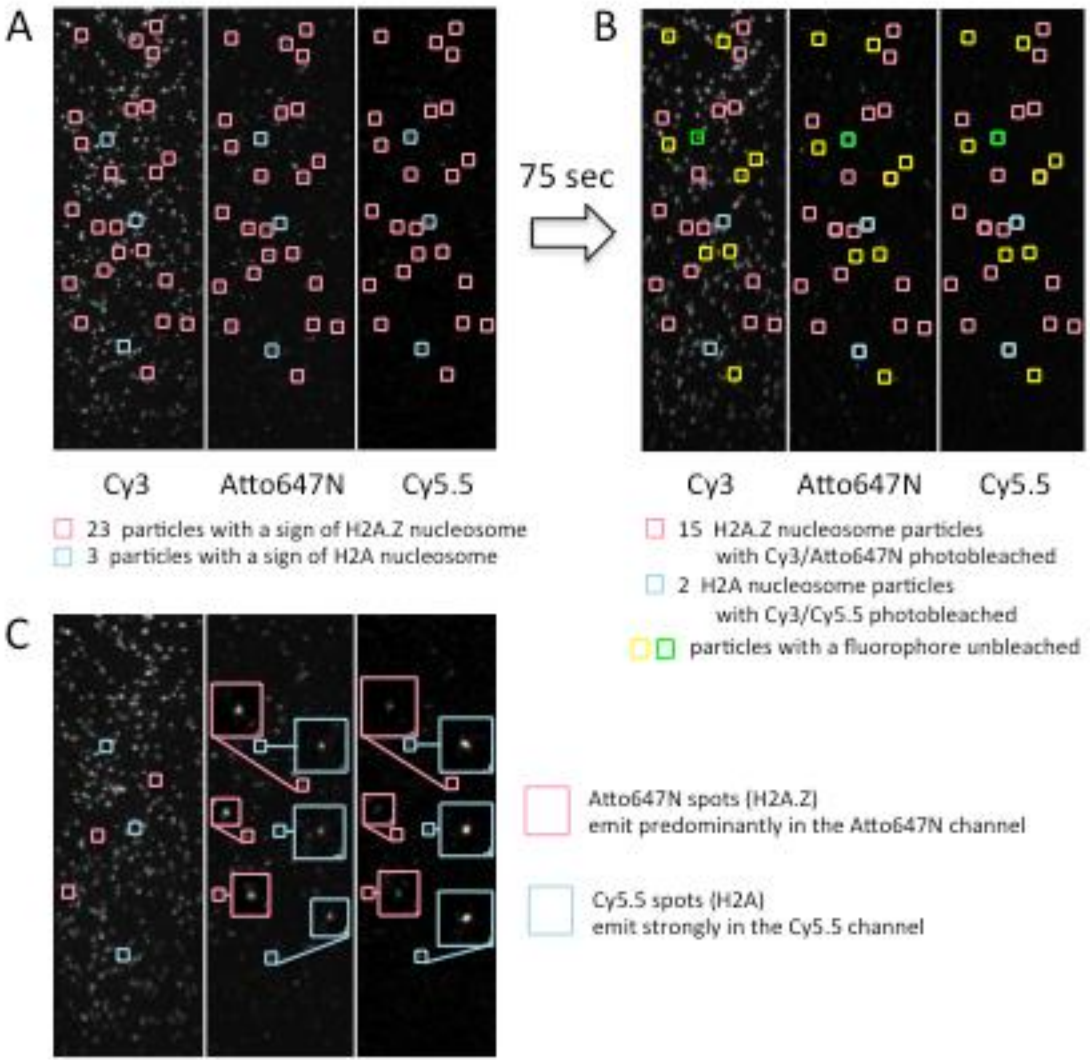

Supplementary Figure 5. Typical screen captures of a single molecule measurement

(a) Image taken at the beginning of a movie recording after 30 min incubation with INO80. **(b)** Image taken at the end of the recording. **(c)** Copy of image (a) with some fluorescent spots magnified. Image (A) shows nucleosome particles emitting acceptor (Atto647N or Cy5.5) fluorescence in the Atto647N channel. Image (b) shows far less particles emitting fluorescence in the Atto647N channel due to acceptor photobleaching. The number of particles that passed our screening criteria (see Methods) constituted on average, 16 % of the total nucleosomes detected in the Atto647N channel at the

beginning of a measurement. This is 64 % of the nucleosomes that are properly labeled for the measurement (i.e. 16 % = 64 % of the 25 % that has only the proximal-dimer labeled according to the 1:1 labeled:unlabeled dimer ratio in the sample). The exact numbers of analyzed particles are given in Figure 5. Note that Cy5.5 emission leaks significantly to the Atto647N channel. Due to this leakage, we are able to detect all the nucleosomes regardless of the dimer identity from the Atto647N channel, which is demonstrated with six nucleosome particles marked in (c). The blue-marked spots in (a) and (c) show a typical Cy3-Cy5.5 FRET pair (H2A nucleosomes, or nucleosomes with H2A.Z replaced by H2A). After 75 sec Cy5.5 is photobleached and strong Cy3 emission is detected in one of the three particles (green-marked spot in (b)). The other two particles have both Cy3 and Cy5.5 photobleached (i.e. no detectable spot in the blue rectangles in (b)), and thus, are qualified for further analysis. The red-marked spots in (a) and (c) show typical Cy3-Atto647N FRET pairs (H2A.Z nucleosome, or nucleosome with H2A.Z retained). After 75 sec, emission from Cy3 or Atto647N is detected in 8 of the 23 particles (yellow-marked spots in (b)). The other 15 particles have both Cy3 and Atto647N photobleached (i.e. no detectable spot in the red rectangles in (b)), and thus, are qualified for further analysis. Sample time trajectories of the fluorescence intensities (Cy3, Atto647N, and Cy5.5) from qualified particles are given in Figure 5.

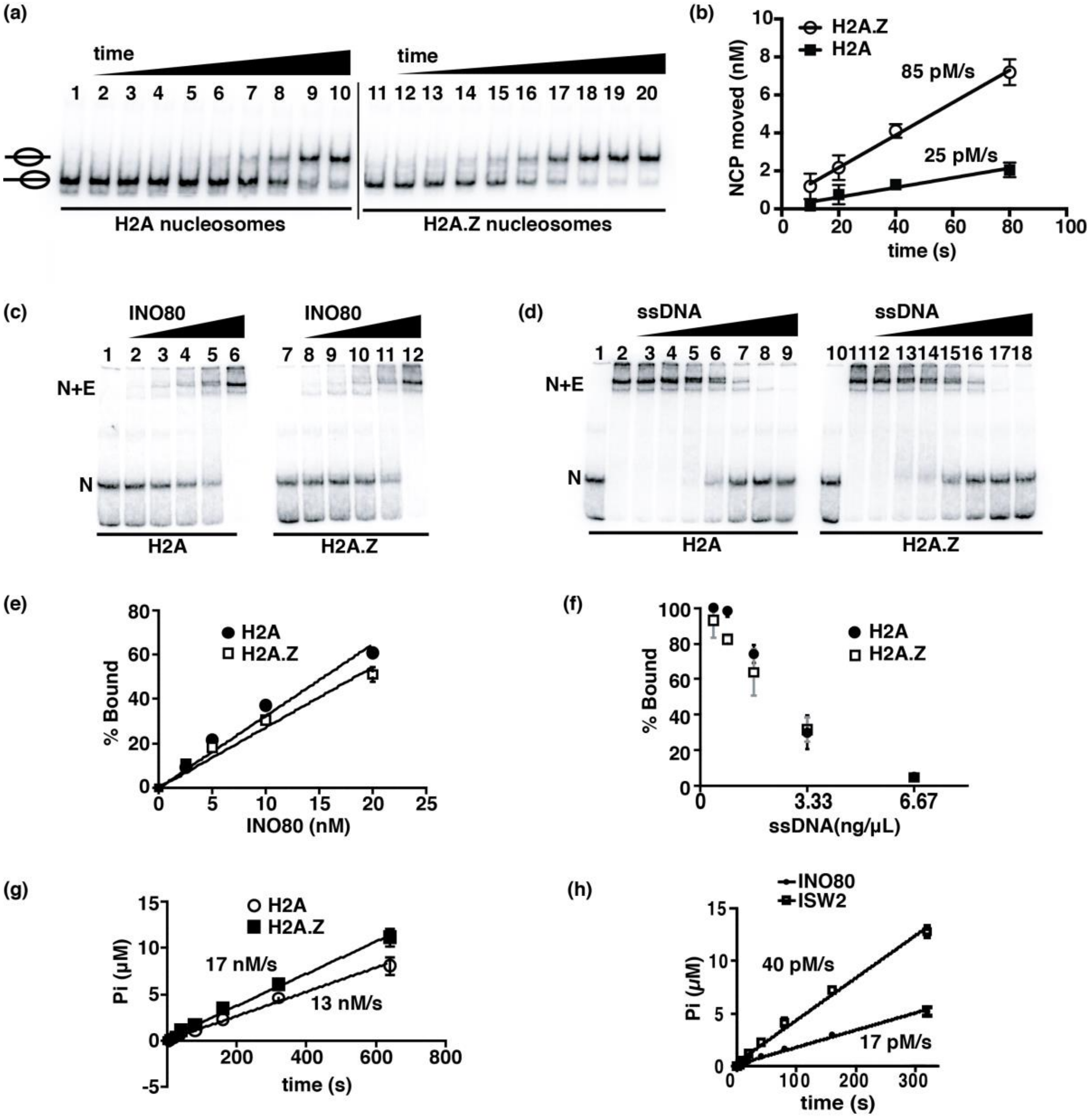

Supplementary Figure 6. INO80 shows H2A.Z specificity for nucleosome remodeling (a) INO80 repositions H2A.Z nucleosomes faster than H2A nucleosomes, as determined by electrophoretic mobility shift in native gel. End-positioned (70N0) nucleosomes containing H2A or H2A.Z were completely bound with INO80 and remodeled with 800 μM ATP at 30ºC for 0, 5, 10, 20, 40, 80, 160, 320, 640, and 1280 s.

Upon remodeling, nucleosomes were moved to more centrally positioned locations resulting in slower migration. **(b)** The amounts of nucleosomes moved were plotted versus time to determine the initial rates of H2A.Z versus H2A nucleosome movement. NCP is for Nucleosome Core Particle. **(c)** Affinity of INO80 for H2A versus H2A.Z nucleosomes was measured by gel shift assays. INO80 (0, 2.5, 5, 10, 20 and 40 nM) was incubated with nucleosomes (40 nM) for 30 min at 30°C. **(d)** Binding conditions were made more stringent by adding increasing amounts of competitor DNA (salmon sperm DNA, 0, 3.1, 6.2, 12.5, 25, 50, 100 and 200 ng/µL) to the reactions containing 40 nM INO80 and 40 nM nucleosomes, and incubated for 30 min at 30°C. **(e-f)** The fractions of nucleosomes bound were plotted versus increasing concentrations of INO80 (e) or ssDNA (f), respectively, from the gels shown in panels c and d. **(g)** Rates of nucleosome-stimulated ATP hydrolysis by INO80 with H2A.Z nucleosomes versus H2A nucleosomes were determined under the same conditions as for remodeling (as in panel a). **(h)** Rates of nucleosome-stimulated ATP hydrolysis by INO80 and ISW2 with H2A nucleosomes were determined by thin layer chromatography and were the same reaction conditions as in the remodeling assays shown in Figure 6c. Data are representative of three technical replicates and error bars represent standard deviation of the mean (SD).

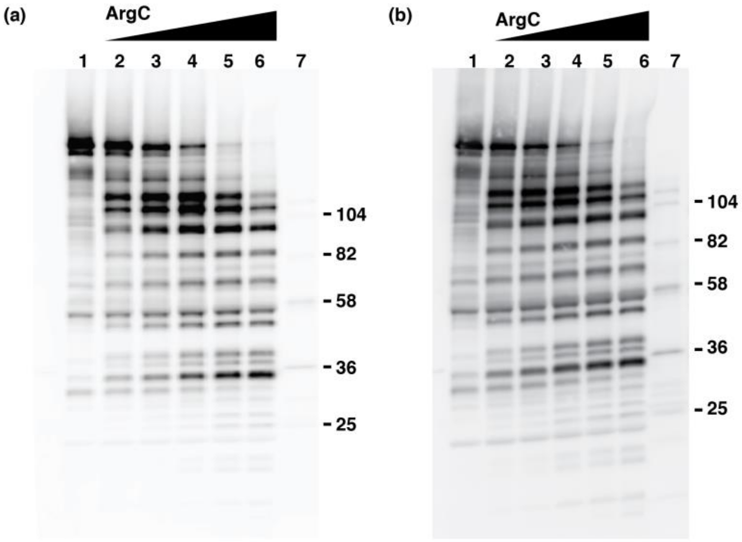

Supplementary Figure 7. Uncropped Western-blot images of Ino80 peptide mappig

(a) Uncropped Western blot from Figure 2d, lanes 1-4 are same as lanes 1-4 in the figure.

(b) Uncropped Western blot from Supplementary Figure 1e, lanes 1-4 are same as lanes 1-4 in the figure.

SUPPLEMENTARY REFERENCES

1. Shen, X. Preparation and analysis of the INO80 complex. *Methods Enzymol* **377**, 401-12 (2004).
2. Tsukiyama, T., Palmer, J., Landel, C.C., Shiloach, J. & Wu, C. Characterization of the imitation switch subfamily of ATP-dependent chromatin-remodeling factors in Saccharomyces cerevisiae. *Genes Dev* **13**, 686-97 (1999).
3. Lowary, P.T. & Widom, J. New DNA sequence rules for high affinity binding to histone octamer and sequence-directed nucleosome positioning. *J Mol Biol* **276**, 19-42 (1998).
4. Kelley, L.A., Mezulis, S., Yates, C.M., Wass, M.N. & Sternberg, M.J.E. The Phyre2 web portal for protein modeling, prediction and analysis. *Nat. Protocols* **10**, 845-858 (2015).
5. Wei, S., Falk, S.J., Black, B.E. & Lee, T.-H. A novel hybrid single molecule approach reveals spontaneous DNA motion in the nucleosome. *Nucleic Acids Research* **10.1093/nar/gkv549**(2015).
6. Lee, J.Y., Lee, J., Yue, H. & Lee, T.H. Dynamics of nucleosome assembly and effects of DNA methylation. *J Biol Chem* **290**, 4291-303 (2015).
